# The Effect of (-)-Epigallocatechin-3-Gallate on IL-1β Induced IL-8 Expression in Orbital Fibroblast from Patients with Thyroid-Associated Ophthalmopathy

**DOI:** 10.1371/journal.pone.0148645

**Published:** 2016-02-05

**Authors:** Ji-Young Lee, Ji-Sun Paik, Mihee Yun, Seong-Beom Lee, Suk-Woo Yang

**Affiliations:** 1 Department of Ophthalmology and Visual Science, Seoul St. Mary’s Hospital, College of Medicine, The Catholic University of Korea, Seoul, Korea; 2 Department of Pathology, College of Medicine, The Catholic University of Korea, Seoul, Korea; Charite Universitätsmedizin Berlin, GERMANY

## Abstract

Orbital fibroblasts have been reported to be an important effector cells for the development of thyroid-associated ophthalmopathy (TAO). Orbital fibroblasts secrete various inflammatory cytokines in response to an inflammatory stimulation, leading to TAO-related tissue swelling. It has also been reported that (-)-epigallocatechin-3-gallate (EGCG), a major polyphenolic constituent of green tea, has antioxidant and anti-inflammatory properties. In the current study, we investigated the issue of whether or how EGCG affects the interleukin (IL)-1β-induced secretion of IL-8 in human orbital fibroblasts from TAO patients. Treatment with EGCG significantly reduced the level of IL-1β-induced secretion of IL-8 and the expression of IL-8 mRNA. IL-1β-induced the degradation of IκBα, and the phosphorylation of p38 and ERK, and the IL-1β-induced expression of IL-8 mRNA was inhibited by specific inhibitors, such as BAY-117085 for NF-kB, SB203580 for p38, and PD98059 for ERK. In addition, treatment with EGCG inhibited the IL-1β-induced degradation of IκBα, and the phosphorylation of p38 and ERK. However, pre-treatment with antioxidants, NVN and NAC, which suppressed ROS generation, did not reduce IL-8 expression in IL-1β-treated orbital fibroblasts, suggesting that the IL-1β-induced IL-8 expression is not mediated by the generation of ROS. These results show that EGCG suppresses the IL-1β-induced expression of IL-8 through inhibition of the NF-κB, p38, and ERK pathways. These findings could contribute to the development of new types of EGCG-containing pharmacological agents for use in the treatment of TAO.

## Introduction

Thyroid-associated ophthalmopathy (TAO), a well-known autoimmune disease, occurs in 25–50% of patients with Graves’ disease [1,2]. The main clinical features of TAO, which include upper eyelid retraction, edema, and erythema of the eyelid, periorbital tissues and conjunctiva, as well as exophthalmos, are mainly due to swelling of the fatty and muscular orbital tissues. These edematous changes in TAO patients are caused by the infiltration of inflammatory cells, the accumulation of extracellular matrix (ECM), the proliferation of fibroblasts and an increased amount of fatty tissue [3].

Significantly higher levels of interleukin (IL)-1β, IL-6, and IL-8 have been observed in primary orbital tissue cultures of TAO patients compared to those of non-TAO patients [4,5]. Hiromatsu et al. [6] reported that that orbital volume was positively correlated with the level of IL-6 mRNA in orbital tissues, showing the importance of IL-6 in the pathogenesis of TAO. In addition, IL-8, a pro-inflammatory cytokine, has also been reported to be associated with the development of TAO [7]. The serum levels of IL-8 have been reported to be associated with the development of Graves’ disease [7]). IL-8 not only recruits neutrophils and T lymphocytes but also promotes the adhesion of immune cells to the endothelial surface [8].

It has been reported that orbital fibroblasts are important effector cells for the development of TAO [9]. Orbital fibroblasts have been reported to secrete IL-6 or/and IL-8, in response to various stimuli, including IL-1β [10], tumor necrosis factor-ɑ (TNF-ɑ) [11], prostaglandin E2 (PGE_2_) [12], platelet-derived growth factor (PDGF-BB) [13], palmitate [14], and cluster of differentiation (CD)154 [15].

The natural product, (-)-epigallocatechin-3-gallate (EGCG) is the major polyphenolic constituent found in green tea which is produced from *Camellia sinensis*. EGCG has been proven to have antioxidant and anti-inflammatory properties in various cells, including fibroblasts [16–18], cardiomyoblasts [19], and cancer cells [20–23].

However, as of this writing, the effect of EGCG on orbital fibroblasts in the pathogenesis of TAO has not been investigated. If EGCG were to have a definitive effect in orbital fibroblasts, it would facilitate alternate therapeutic approaches to the treatment of TAO. In the current study, we investigated the issue of whether or how EGCG affects the IL-1β-induced secretion of IL-8 in human orbital fibroblasts obtained from TAO patients.

## Materials and Methods

### Reagents and antibodies

(-)-epigallocatechin-3-gallate (EGCG), N-acetylcysteine (NAC), and N-vanillylnonanamide (NVN) were obtained from Sigma Aldrich Co. Ltd. (St. Louis, MO). Recombinant human IL-1β was purchased from GIBCO BRL (Grand Island, NY). The inhibitors, BAY 11–7085 for NF-κB, SB 203580 for p38, and PD 98059 for MEK 1, were purchased from Calbiochem (La Jolla, CA). EGCG and NAC were dissolved in H_2_O. NVN, BAY 11–7085, SB 203580, and PD 98059 were dissolved in dimethyl sulfoxide, methyl alcohol, or H_2_O. The final vehicle concentration was adjusted to 0.1% (v/v), and the control medium contained the same quantity of vehicle. Antibodies against phospho-p38, p38, phospho-ERK, and ERK were obtained from Cell Signaling Technology (Beverly, MA). Antibodies against IκBα, β-tubulin, horseradish peroxidase, and Cy3-conjugated secondary antibodies were obtained from Santa Cruz Biotechnology (Santa Cruz, CA).

### Cell culture

Human orbital fibroblasts were collected from the orbital fat obtained from patients with TAO who had undergone decompression surgery or from upper lid blepharoplasties from patients with no prior history of thyroid disease, TAO, and no clinical evidence of inflammatory or immune diseases. Before the decompression surgery, all patients with TAO had experienced at least 6 months of inactive disease status with a euthyroid condition (Table 1). Orbital fat explants were minced in small pieces, attached to plastic culture dishes, and covered with Dulbecco’s Modified Eagle’s medium (GIBCO BRL, Grand Island, NY) supplemented with 20 mM HEPES (Fisher Scientific, Atlanta, GA), 10% fetal bovine serum (FBS; GIBCO BRL), 100 U/mL of penicillin, and 100 μg/mL of streptomycin (Bio Whittaker Inc., Walkersville, MD). Non-adherent cells and fat tissue was then removed, and the established fibroblasts were passaged with gentle trypsin/EDTA treatment. Fibroblasts were not used for studies beyond passage 10 from the initial culture. These activities were undertaken after written informed consent was obtained from the donors, according to procedures approved by the Institutional Review Board of Seoul St. Mary’s Hospital (KC10TISE0743) and the tenets of the Declaration of Helsinki.

**Table 1:** Characteristics of patients with thyroid-associated ophthalmopathy (TAO) and subjects without TAO from whom the experimental orbital fibroblasts were obtained.

	Patients with TAO	Subjects without TAO
	(n = 4)	(n = 4)
**Mean age, years (range)**	46.5 (23–63)	53.3 (31–69)
**Sex (m/f)**	2/2	1/3
**Smoking (years)**	1	1
**Graves’ dis**		0
**Radioactive iodine therapy**	1	-
**Surgery**	0	-
**Methimazole**	4	-
**Treatment of TAO**		0/4
**Surgery**	4	-
**Prednisolone**	3	-
**Radiation**	1	-
**Euthyroid**	4	4
**TSH receptor antibodies**	4	0
**CAS (range)**	3 (2–4)	(-)

CAS: clinical activity score

### Inhibitor treatments

Human orbital fibroblasts were plated onto 6-well plates or 24-well plates. After 24 hours, the cells were pre-treated with EGCG, NVN, NAC, BAY 11–7085, SB 203580, or PD 98059 for 1 hour, followed by treatment with IL-1β (10 ng/mL) for the indicated times. After incubation for the designated times, the cells were harvested and used in subsequent experiments. The following concentrations of inhibitors were used: EGCG, 10–100 μM; NVN, 0.2 mM; NAC, 5 mM; BAY 11–7085, 1 and 5 μM; SB 203580, 10 and 20 μM; PD 98059, 10 and 20 μM. None of the inhibitors used had a significant effect on the viability of the human orbital fibroblasts.

### Cell viability assay

Cell viability was measured using the 3-(4,5-dimethylthiazol-2-yl)-2,5-diphenyltetrazolium bromide (MTT) reduction assay in 96-well plates. Briefly, at designated times, 10 μL of an MTT solution (5 mg/mL) was added to each well. After incubation in a 5% CO_2_ incubator for 2 hours at 37°C, the media was removed, and 100 μL of acidified isopropyl alcohol was added to each well. After 10 minutes of incubation, 100 μL of distilled water was added to each well, and then the optical density of each well was then read by a spectrometer at a wavelength of 570 nm. Data are expressed as the percentage of the untreated control cells.

### Measurement of intracellular ROS

Intracellular ROS levels were measured using the fluorescent probe H_2_DCF-DA, which, after crossing the plasma membrane was deacetylated to H_2_DCF and oxidized to the fluorescent product DCF. Human orbital fibroblasts were cultured for 24 hours in 96-well black plates, washed with Hanks' Balanced Salt Solution buffer (HBSS, Sigma-Aldrich), and incubated for 1 hour at 37°C in HBSS containing H_2_DCF-DA (10 μM). The medium was changed to fresh HBSS containing EGCG (10–100 μM), NVN (0.2 mM) and NAC (5 mM) for 1 hour at 37°C, and then the cells were then stimulated with or without IL-1β (10 ng/mL) for 30 minutes. The fluorescence intensity was measured using a Fluorometer (Victor 3 Multilabel counter system; Perkin Elmer, Waltham, MA) with excitation at 485 nm and emission at 535 nm.

### Western blot analysis

Human orbital fibroblasts were cultured to confluence and treated for 1 hour with or without a designated inhibitor, followed by treatment with IL-1β (10 ng/mL) for 10 minutes. The treated cells were removed from the incubator at the indicated times, placed on ice, and washed three times with ice-cold PBS. The cells were then lysed for 30 minutes with RIPA lysis buffer [50 mM Tris-HCl [pH 7.4], 1% Triton X-100, 150 mM NaCl, 0.1% sodium dodecyl sulfate (SDS), 0.5% sodium deoxycholate, 100 mM phenylmethylsulfonyl fluoride, 1 μg/mL of leupeptin, 1 mM Na_3_VO_4_, and 1× Complete^™^ Protease Inhibitor Cocktail (Santa Cruz Biotechnology)]. Equal amounts of protein were loaded onto 10–15% SDS-polyacrylamide gel electrophoresis gels, electrophoresed, and transferred to polyvinylidene difluoride membranes (Millipore, Bedford, MA). The membranes were blocked in Tris-buffered saline with 0.05% Tween-20 (TBST) supplemented with 5% powdered milk or 5% bovine serum albumin, and then incubated with the appropriate primary antibodies. The blots were then washed with TBST and incubated with a horseradish peroxidase-conjugated secondary antibody in TBST plus 5% powdered milk. The bound antibodies were detected with Super Signal Ultra Chemiluminescence Reagents (Pierce Biotechnology, Inc., Rockford, IL).

### Cytokine assays

Human orbital fibroblasts were cultured to confluence and then treated for 1 hour with or without a designated inhibitor, followed by treatment with IL-1β (10 ng/mL) for 24 hours. The culture medium was analyzed for IL-6 and IL-8 contents using a standard sandwich enzyme linked immunosorbent assay (ELISA) kit (R&D Systems, Minneapolis, MN) according to the manufacturer’s instructions.

### Quantitative real time reverse transcription-polymerase chain reaction (RT-PCR)

IL-8 mRNA expression was determined by quantitative real time-RT–PCR. Briefly, total RNA was extracted using a High Pure RNA Isolation kit (Roche Diagnostics, Mannheim, Germany), and converted into cDNA using an Advantage RT-for-PCR kit (Clontech, Hampshire, UK) according to the manufacturer’s instructions. To quantify IL-8 mRNA, quantitative real time RT–PCR was performed using a iQ^™^ SYBR^®^ Supermix kit (Bio-Rad Laboratories, Hercules, CA) in a Peltier Thermal Cycler-200 system (MJ Research, Berlin, Germany). RT-PCR was performed in triplicate at 95°C for 10 minutes followed by 44 cycles of amplification (95°C for 20 seconds, 60°C for 10 seconds, and 72°C for 15 seconds). The relative amount of IL-8 mRNA was determined by subtracting the cycle threshold (Ct) values from the Ct values for 28S rRNA. The following primers for IL-8 and 28S rRNA were used: For IL-8, forward primer (5′-ATA AAG ACA TAC TCC AAA CCT TTC CAC-3′) and reverse primer (5′-AAG CTT TAC AAT AAT TTC TGT GTT GGC-3′). For 28S rRNA, forward primer (5′-ACG GTA ACG CAG GTG TCC TA-3′) and reverse primer (5′-CCG CTT TCA CGG TCT GTA TT-3′).

### Statistical Analysis

All of the results reported herein are expressed as the mean ± standard error of the mean (SEM) of data from at least three separate experiments. Statistical significance was determined via Student’s *t*-test or one-way ANOVA. *p* < 0.01 or 0.05 was considered to be statistically significant.

## Results

### Effect of EGCG on IL-1β-induced IL-8 expression in orbital fibroblasts

**Fig 1:**
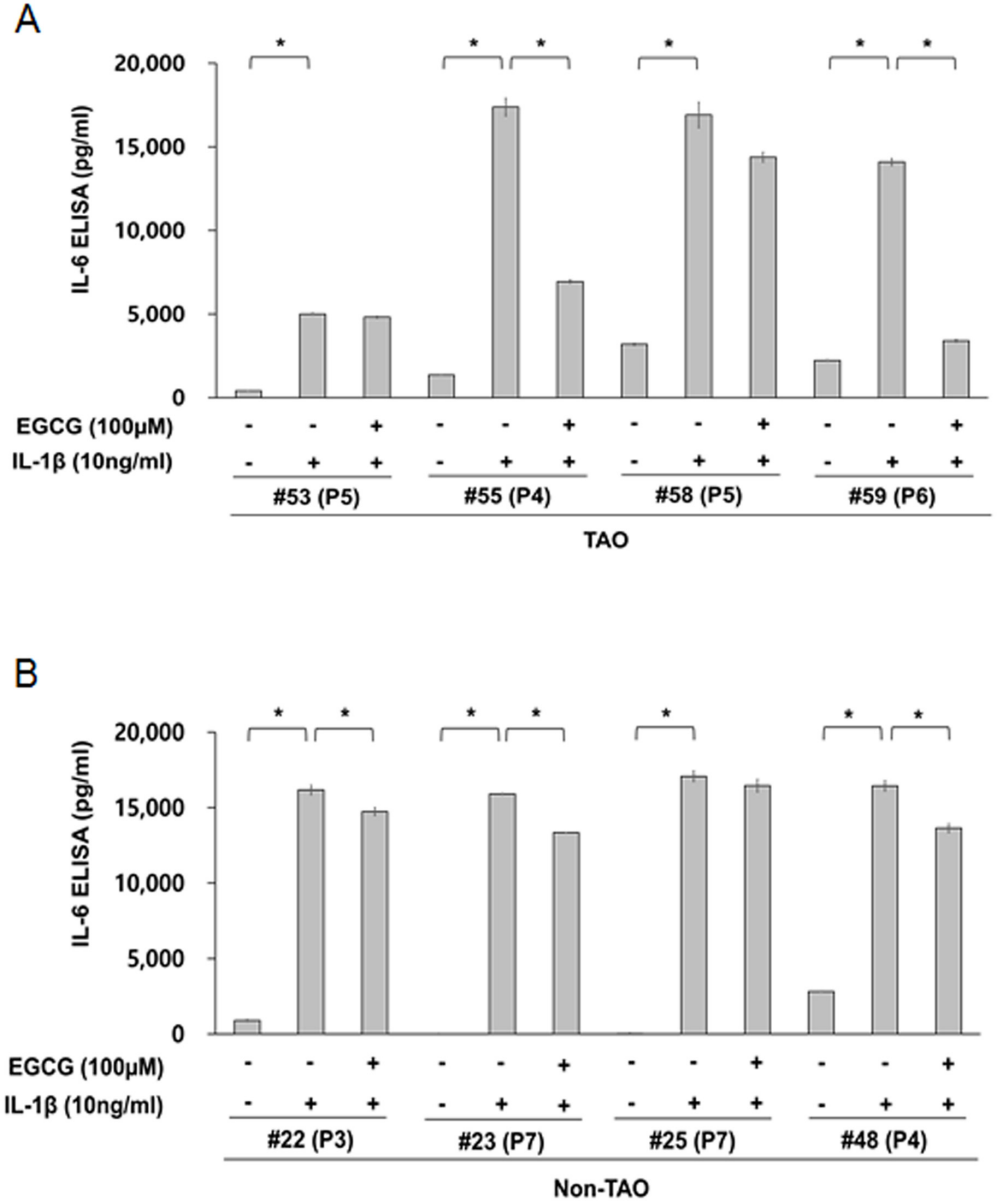
The capability of orbital fibroblasts to secret IL-6 in response to proinflammatory cytokines, IL-1β. Orbital fibroblasts were pre-treated with or without EGCG for 1 h, followed by treatment with IL-1β (10 ng/mL) for 24 h. The level IL-6 was determined by ELISA (R&D Systems, Minneapolis, MN). *p < 0.01 between the indicated groups as calculated by Student’s t-test. P, number of passages.

**Fig 2:**
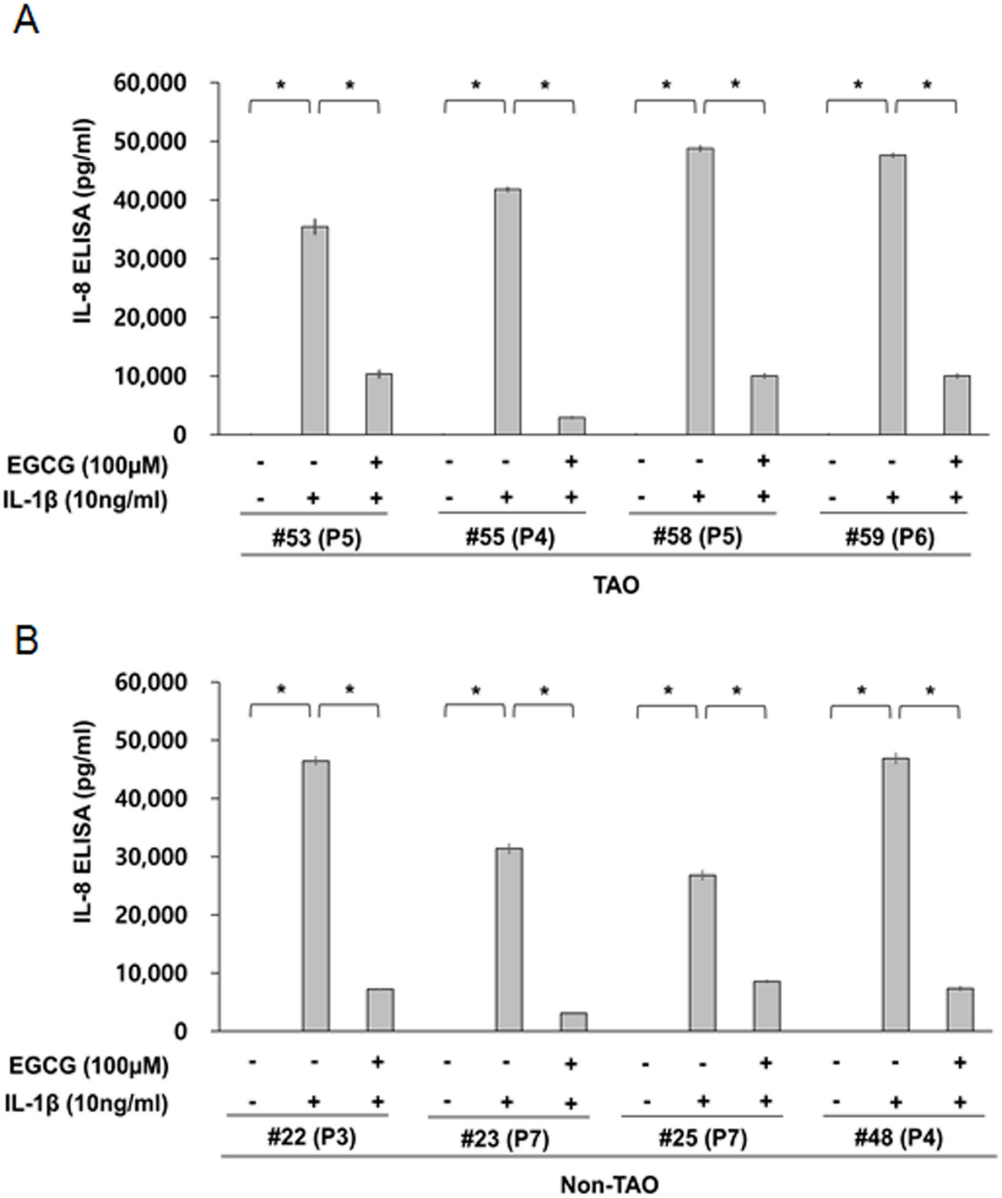
The capability of orbital fibroblasts to secret IL-8 in response to proinflammatory cytokines, IL-1β. Orbital fibroblasts were pre-treated with or without EGCG for 1 h, followed by treatment with IL-1β (10 ng/mL) for 24 h. The level IL-8 was determined by ELISA (R&D Systems, Minneapolis, MN). *p < 0.01 between the indicated groups as calculated by Student’s t-test. P, number of passages.

We initially evaluated the capability of orbital fibroblasts to secrete IL-6 and IL-8 in response to the proinflammatory cytokine, IL-1β. Depending on the strains of orbital fibroblasts, they show different capabilities for secreting IL-6 and IL-8, under basal conditions and in response to IL-1β. The basal level of IL-6 in orbital fibroblasts from patietns with TAO (range: 405–3,195 pg/ml, mean: 1,794 pg/ml) and non-TAO (range: 0–2,807 pg/ml, mean: 925 pg/ml) patients varied depending on the strains of orbital fibroblasts (Fig 1), whereas IL-8 was not detected in non-treated orbital fibroblasts from either groups (Fig 2). The levels of IL-1β-induced IL-6 secretion were similar in orbital fibroblasts from all patients (mean: 16,291 pg/ml), except for TAO patient # 53 (mean: 5,004 pg/ml), regardless of whether the patients had TAO or not (Fig 1). However, the levels of IL-1β-induced IL-8 secretion varied depending on the strains of orbital fibroblasts from TAO (range: 35,430–48,770 pg/ml, mean: 43,402 pg/ml) and non-TAO patients (range: 26,805–46,872 pg/ml, mean: 37,885 pg/ml) (Fig 2).

We also examined the effect of EGCG on IL-1β-induced IL-6 and IL-8 secretion in orbital fibroblasts. A pre-treatment with EGCG consistently reduced the level of IL-1β-induced secretion of IL-8 by 68.0–93.0% in orbital fibroblasts from patietns with TAO and non-TAO (Fig 2). However, there was significant variation in the level of reduction of IL-1β-induced secretion of IL-6 by EGCG within the strains of oribtal fibroblasts. Pre-treatment with EGCG reduced the secretion of IL-6 in IL-1β-treated orbital fibroblasts slightly, in all patients with non-TAO (reduction level: range: 3.7–17.0%, mean: 11.4%, Fig 1B) and two patients with TAO (reduction level: 3.8% in #53 and 15.2% in #58)(Fig 1A), whereas a significant inhibitory effect of EGCG on IL-6 secretion was observed in orbital fibroblasts form two TAO patients (reduction level: 60.0% in #55 and 75.9% in #59)(Fig 1A). However, an evaluation of these discrepancies in the inhibitory effect of EGCG on IL-6 secretion, between the cell strains of TAO patients or non-TAO patients, was beyond the scope of the current study. Thus, we restricted our study to the inhibitory effect of EGCG on IL-8 secretion in orbital fibroblasts of TAO patients.

**Fig 3:**
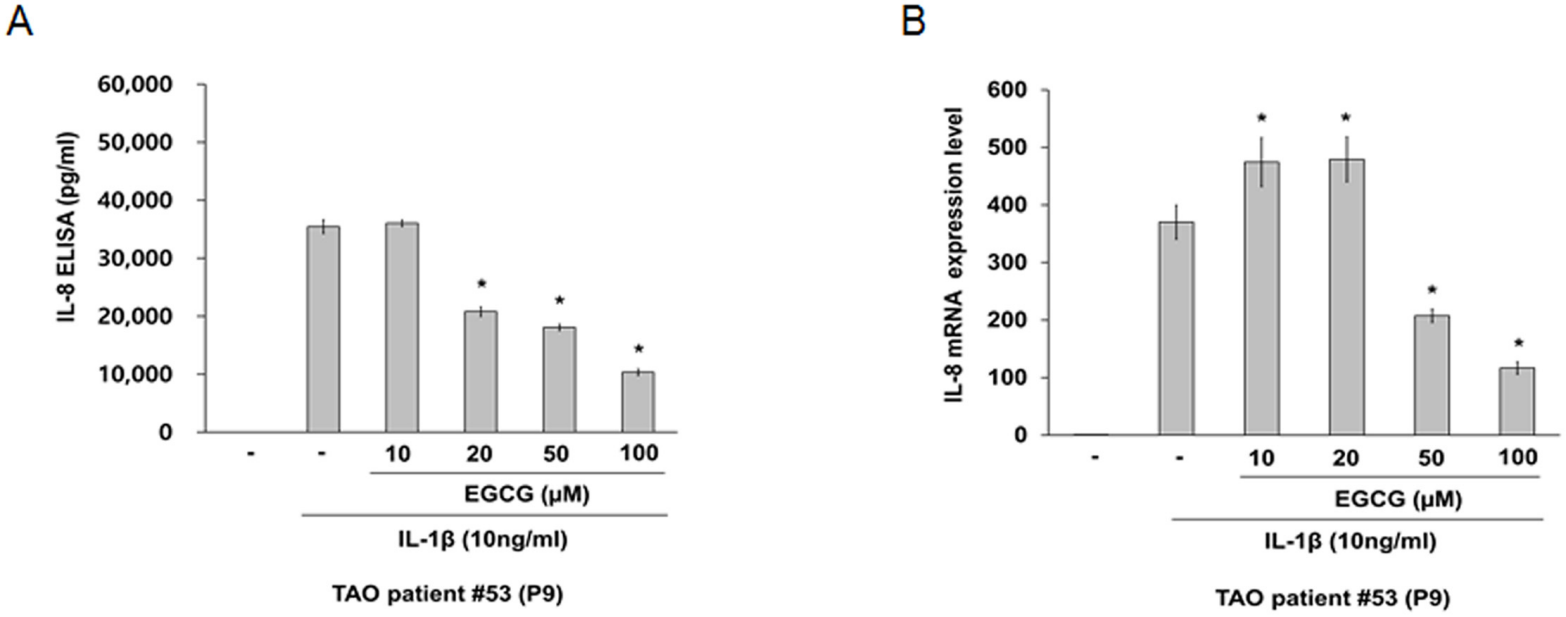
Effect of EGCG on IL-1β-induced IL-8 expression in orbital fibroblasts. Orbital fibroblasts were pre-treated with EGCG for 1 h, followed by treatment with IL-1β (10 ng/mL) for 24 h. (A) The level of IL-8 was determined using by ELISA (R&D Systems, Minneapolis, MN). Similar results were obtained in three independent experiments with orbital fibroblasts of passages (P) 9 and P10 from TAO patient #53 and P4 from TAO patient #55. (B) The IL-8 mRNA levels were determined by quantitative real time RT-PCR). *p < 0.05 compared with cells treated with the same concentration of IL-1β as calculated by one-way ANOVA. Similar results were obtained in four independent experiments with orbital fibroblasts of P7, P9, and P10 from TAO patient #53 and P4 from TAO patient #55. P, number of passages.

As shown in Fig 3, a pre-treatment with EGCG, which has no significant cytotoxic effect on the viability of orbital fibroblasts at 10–100 μM (S1 Fig), significantly reduced the level of IL-1β-induced IL-8 secretion in a dose dependent manner (A) and expression of IL-8 mRNA at 50 and 100 μM (B).

### Effect of antioxidants on IL-1β-induced IL-8 Secretion in Orbital Fibroblasts

**Fig 4:**
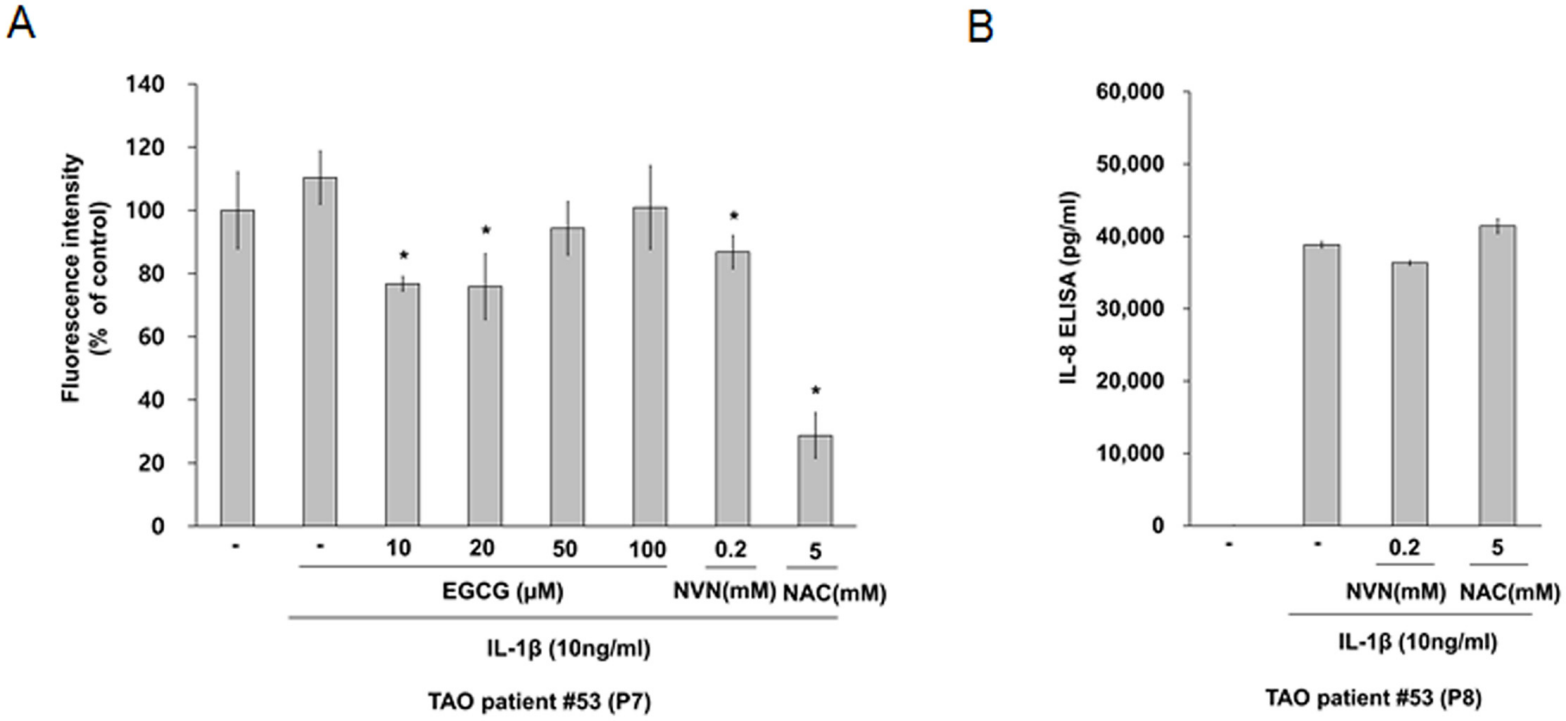
Effect of antioxidants on IL-1β-induced IL-8 secretion in orbital fibroblasts. Orbital fibroblasts were pre-treated with EGCG, NVN, and NAC for 1 hour, followed by treatment with IL-1β (10 ng/mL) for 30 minutes. (A) The intracellular levels of ROS using H2DCF-DA were measured by fluorometry Similar results were obtained in three independent experiments with orbital fibroblasts of passages (P)7 and P9 from TAO patient #53. (B) Orbital fibroblasts were pre-treated with NVN and NAC for 1 hour, followed by treatment with IL-1β (10 ng/mL) for 24 hours. IL-8 levels were determined by ELISA (R&D Systems, Minneapolis, MN). Similar results were obtained in three independent experiments with orbital fibroblasts of P8 and P9 from TAO patient #53. *p < 0.05 compared with cells treated with the same concentration of IL-1β as calculated by one-way ANOVA. P, number of passages.

It has been reported that IL-1β induces IL-8 expression via the activation of mitogen activated protein kinase (MAPK) and the generation of ROS in human gastric adenocarconoma cells [15]. EGCG is a well-known antioxidant. Pre-treatment with EGCG at concentrations of 10 μM and 20 μM reduced IL-1β-induced ROS production, as shown in cells that had been pre-treated with other antioxidants (NVN and NAC; Fig 4A). However, pre-treatment with the antioxidant NVN or NAC did not reduce IL-1β-induced IL-8 secretion in orbital fibroblasts (Fig 4B). These results suggest that IL-1β-induced IL-8 secretion is not mediated by the ROS generation in orbital fibroblasts.

### Role of NF-κB, p38, and ERK in the Regulation of IL-1β-induced IL-8 Expression in Orbital Fibroblasts

**Fig 5:**
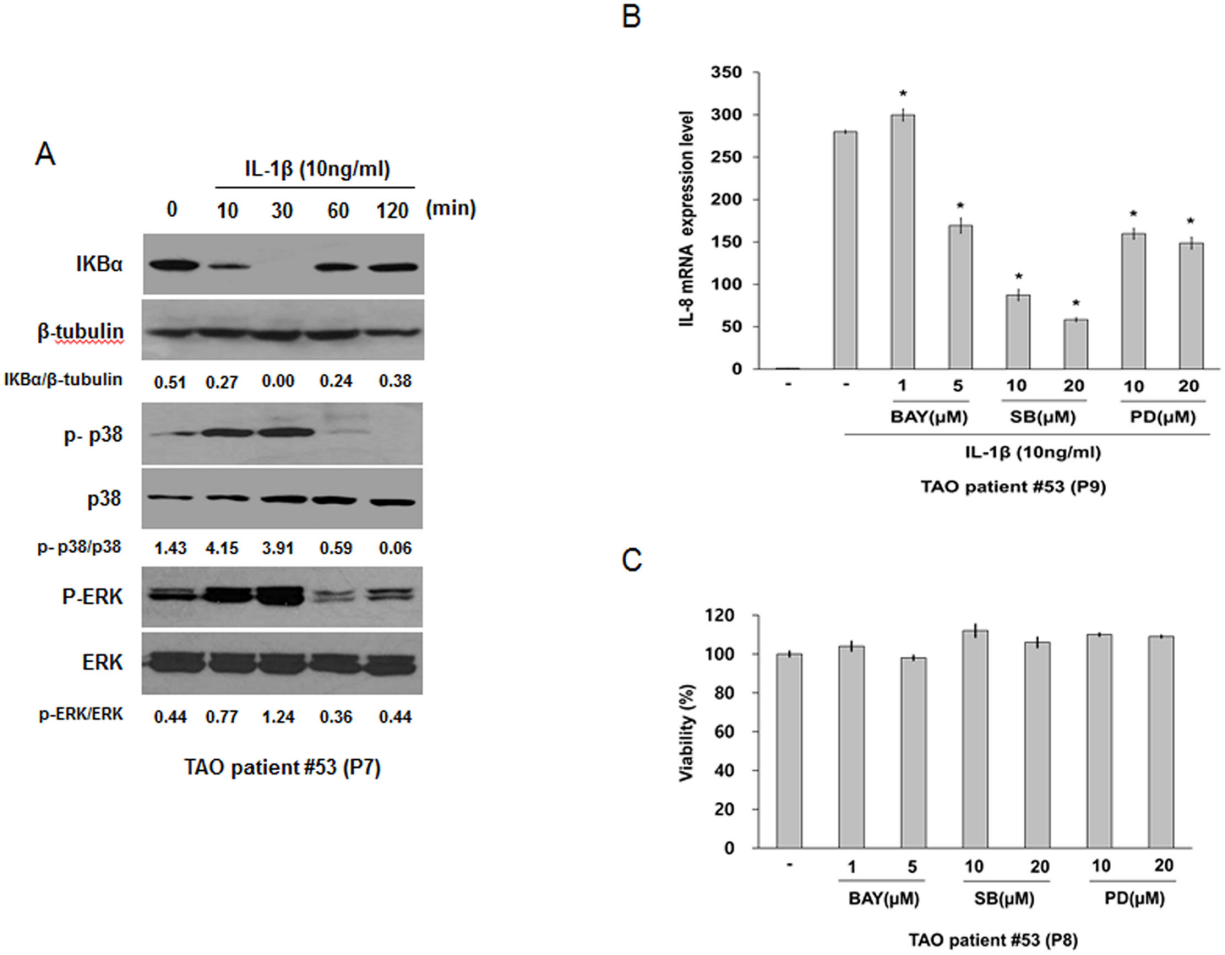
Effect of IL-1β on the NF-κB, p38, and ERK pathways in orbital fibroblasts, and the role of the NF-κB, p38, and ERK pathways in IL-1β-induced IL-8 expression in orbital fibroblasts. (A) Orbital fibroblasts were treated with IL-1β (10 ng/mL) for 10–120 minutes. At the designated times, the degradation of IκBα and the phosphorylation of p38 and ERK were confirmed by Western blot analysis. Similar results were obtained in three independent experiments with orbital fibroblasts of passages (P)7 and P10 from TAO patient #53. (B) Orbital fibroblasts were pre-treated with inhibitors, BAY 11–7085 for NF-κB, SB 203580 for p38, or PD 98059 for ERK for 1 hour, followed by treatment with IL-1β (10 ng/mL) for 24 hours. IL-8 mRNA level was determined by quantitative real time RT-PCR. Similar results were obtained in three independent experiments with orbital fibroblasts of P9 and P10 from TAO patient #53. (C) Orbital fibroblasts were treated with BAY 11–7085, SB 203580, or PD 98059 for 24 hours. Cell viability was measured using the MTT reduction assay. Similar results were obtained in three independent experiments with orbital fibroblasts of P8 and P10 from TAO patient #53. *p < 0.05 compared with cells treated with the same concentration of IL-1β as calculated by one-way ANOVA. P, number of passages.

**Fig 6:**
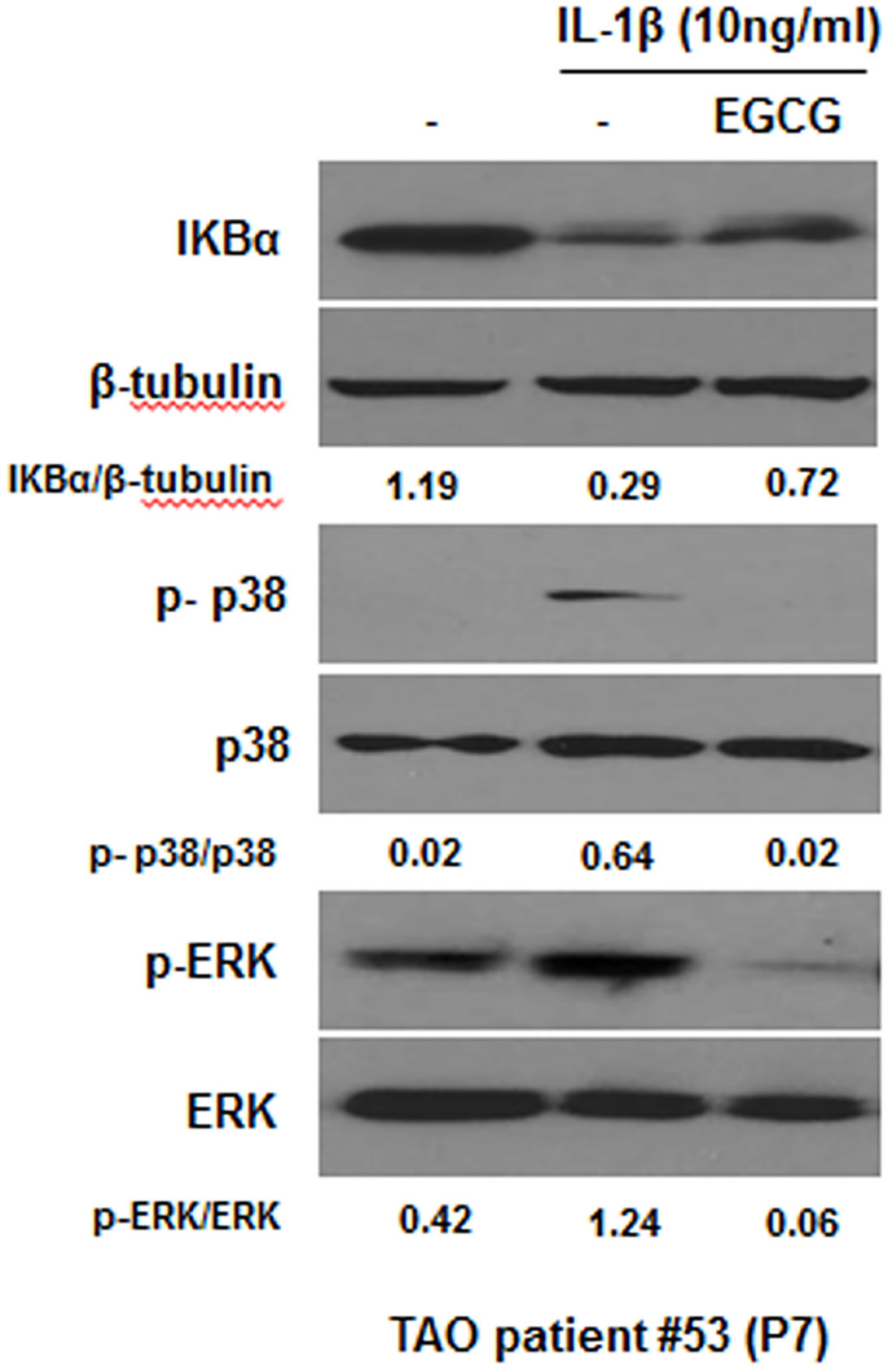
Effect of EGCG on IL-1β induced-degradation of IκBα and -phosphorylation of p38 and ERK in orbital fibroblasts. Orbital fibroblasts were pre-treated with EGCG (100 μM) for 1 hour, followed by treatment with IL-1β (10 ng/mL) for 10 minutes. The degradation of IκBα and the phosphorylation of p38 and ERK were confirmed by Western blot analysis. Similar results were observed in three independent experiments with orbital fibroblasts of passages (P)7 and P10 from TAO patient #53. P, number of passages.

We investigated the issue of whether IL-1β-induced IL-8 expression is mediated by the NF-κB, p38, and ERK pathways in orbital fibroblasts. We initially measured the levels of IκBα degradation, phosphorylated p38 and ERK in IL-1β-treated orbital fibroblasts by immunoblot analysis. Treatment with IL-1β (10 ng/mL) induced the degradation of IκBα, the phosphorylation of p38 and ERK in orbital fibroblasts at 10 and 30 minutes (Fig 5A). We then examined the role of NF-κB, p38, and ERK in the IL-1β-induced expression of IL-8 in orbital fibroblasts using a specific inhibitor for NF-κB, p38, or ERK. Pre-treatment with the specific inhibitors such as BAY-117085 for NF-kB, SB203580 for p38, and PD98059 for ERK, at an inhibitor concentration that has no significant cytotoxic effect on the viability of orbital fibroblasts (Fig 5C), inhibited IL-1β-induced IL-8 expression at the mRNA level (Fig 5B). We next examined the effect of EGCG on the NF-κB, p38, and ERK pathways. As shown in Fig 6, pre-treatment with EGCG suppressed the IL-1β-induced degradation of IκBα, and the phosphorylation of p38 and ERK. These results suggest that EGCG suppressed IL-1β-induced IL-8 expression through the inhibition of the NF-κB, p38, and ERK pathways.

## Discussion

TAO is a potentially vision-threatening ocular disease that remains difficult to treat, and its pathologic mechanism is not completely understood at present. Previous reports indicate that orbital fibroblasts serve as the target cells in TAO that recruit T cells, resulting in reciprocal and subsequent tissue remodeling, which is a characteristic of TAO [15,24].

IL-6 and IL-8 have been reported to be strongly associated with the development of TAO [6,7]. Orbital fibroblasts are the major source of IL-6 and IL-8 and play an important role in the development of TAO [9]. The findings reported herein show that the basal level of IL-6 varied depending on the strains of orbital fibroblasts obtained from TAO or non-TAO patients (Fig 1) and that there was no significant difference in the basal level of IL-6 between TAO and non-TAO patients (Fig 1). Consistent with these results, Hwang et al. [15] and van Steensel et al. [13] also reported that the basal levels of IL-6 and IL-8 in orbital fibroblasts were not significantly different between TAO patients and control patients without any known thyroid disease.

In terms of stimuli responsiveness of orbital fibroblasts, our results also showed that no significant differences in the level of IL-6 and IL-8 from IL-1β-treated orbital fibroblasts were observed between TAO and non-TAO patients, although orbital fibroblasts show different capabilities for secreting IL-6 and IL-8 in response to IL-1β depending on the strains of orbital fibroblasts (Figs 1 and 2). However, Hwang et al. [15] reported that several strains of TAO fibroblasts secret higher levels of IL-8, but similar levels of IL-6, in response to IL-1β than control cells. Although it is difficult to directly compare our results with results reported by Hwang et al. [15] and van Steensel et al. [13] due to differences in the status of the TAO patients and experimental conditions, the reasons for these discrepancies would be helpful in terms of assessing whether or how long fibroblasts-derived from orbital fat of TAO patients maintain the capability to secret inflammatory cytokines during continuous passaging.

There are several types of catechin derivatives in extracts, including (-)-epigallocatechin-3-gallate (EGCG), (-)-epicatechin-3-gallate (ECG), (-)-epigallocatechin (EGC), (-)-epicatechin (EC) and (+)-catechin. EGCG accounts for more than 50% of the mass of this catechin combination. Previous studies reported that EGCG in green tea has very strong antioxidant activity [25–27] and anti-inflammatory effects [16,23,28]. Li et al. [25] reported that EGCG inhibits the effects of inflammation by interfering with ROS generation in macrophages. In addition, Ko et al. [29] previously reported that ROS mediated IL-1β-induced IL-8 expression in Down syndrome candidate region-1-overexpressed human embryonic kidney (HEK) 293 cells. However, our results show that IL-1β-induced IL-8 expression is not mediated by the generation of ROS in orbital fibroblasts (Fig 4B). The reason for such a discrepancy is presently unclear, but could be due to differences in cell type (orbital fibroblasts vs. HEK cells) and experimental methods. Furthermore, ROS generation stimulated by IL-1β in orbital fibroblasts did not appeare to be as significant as in previous reports, as shown in Fig 4A.

The findings reported herein indicate that EGCG inhibits chemokine secretion in orbital fibroblasts from patients with TAO, and in particular, the decrease in IL-8 secretion was statistically signifiant and the mechanisms responsible for this can be attributed to the suppression of the NF-κB, and MAPK pathways, including the p38, and ERK pathway. Consistent with our results, Hwang et al. [30] reported that IL-1β stimulated the expression of IL-8 via the p38 and ERK pathways in human gastric carcinoma cells. Inokawa et al. [31] also reported that NF-κB inhibiton reduced the IL-1β-induced secretion of IL-8 and MCP-1 in human corneal fibroblasts.

In conclusion, our results showed that EGCG, a major constituent of green tea extract, has antioxidant properties, and has a clear anti-inflammatory effect on orbital fibroblasts. The anti-inflammatory effect of EGCG is associated with a reduction in the IL-1β-induced secretion of IL-8. In addition, the observed effects of EGCG on the IL-1β-induced secretion of IL-8 are probably not specific to the orbital fibroblasts from patients with TAO, but rather generally occur in fibroblasts that are exposed to inflammatory conditions. Therefore, it would appear reasonable to recommend an increased intake of green tea or a green tea extract rich in EGCG for patients with active inflammatory TAO or other inflammatory diseases.

These results could contribute to the development of EGCG-containing new pharmacological agent for treating TAO. However, further human clinical studies will be required to establish the beneficial effect of EGCG on TAO, and to identify the therapeutic range of EGCG for use in conjunction with TAO patients.

## Supporting Information

S1 Fig Evaluation of the cytotoxicity of EGCG in orbital fibroblasts of TAO. Orbital fibroblasts were treated with EGCG for 24 hours. Cell viability was measured using the MTT reduction assay. A pre-treatment with EGCG, which has no significant cytotoxic effect on the viability of orbital fibroblasts at 10–100 μM. Similar results were obtained in three independent experiments with orbital fibroblasts of P8 and P10 from TAO patient #53.
(TIF)
