# Supplementary figures and images for: The Effect of (-)-Epigallocatechin-3-Gallate on IL-1β Induced IL-8 Expression in Orbital Fibroblast from Patients with Thyroid-Associated Ophthalmopathy
Source: PLoS One. 2016 Feb 5;11(2):e0148645. doi: 10.1371/journal.pone.0148645 (PMC4743944; doi:10.1371/journal.pone.0148645)

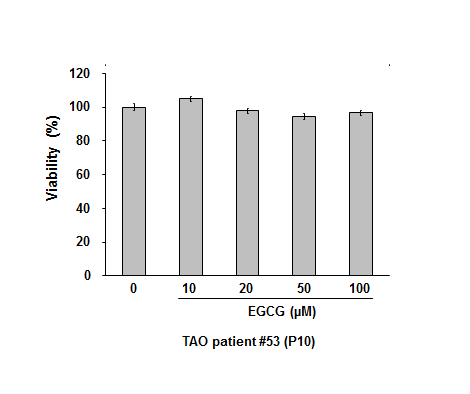

Supplement: S1 Fig — Orbital fibroblasts were treated with EGCG for 24 hours. Cell viability was measured using the MTT reduction assay. A pre-treatment with EGCG, which has no significant cytotoxic effect on the viability of orbital fibroblasts at 10–100 μM. Similar results were obtained in three independent experiments with orbital fibroblasts of P8 and P10 from TAO patient #53. (TIF) [file pone.0148645.s001.tif]
